# Acute Posterior Shoulder Dislocation with Reverse Hill-Sachs Lesion of the Epiphyseal Humeral Head

**DOI:** 10.5402/2011/851051

**Published:** 2011-07-25

**Authors:** Luigi Patrizio, Ettore Sabetta

**Affiliations:** Struttura Complessa Ortopedia e Traumatologia, Arcispedale Santa Maria Nuova, Azienda Ospedaliera di Reggio Emilia, Viale Risorgimento n. 80, 42100 Reggio Emilia, Italy

## Abstract

Posterior dislocation of the shoulder is an unfrequent event that often occurs as a consequence of a direct trauma or epileptic crisis. Frequently the posterior dislocations are misunderstood, so they become chronic lesions. We reported a case of an acute posterior left shoulder dislocation with lesser tuberosity fracture and reverse Hill-Sachs lesions which involved more than 25% of the articular surface of the humeral head, in a 57-old-year man with right hemiparesis. We performed a synthesis of the lesser tuberosity with a screw, and we restored the shape of the humeral head with allograft. We achieved a good result that allows the patient to be able to do his previous activities of daily living.

## 1. Introduction

The causes of posterior shoulder dislocations are usually a **** strong traumatic event or ****a sudden violent internal rotatory muscles contraction such as what may happen during a convulsive crisis or electrocution [1–3].

In most of the cases, the dislocation is subacromial and posterosuperior; the subspinatus and subglenoidea forms are very uncommon.

Anteromedial humeral head impaction fractures (McLaughlin lesion or reverse Hill Sacs) always complete this scenario. Although posterior capsular or labral laceration seems to be constant, posterior tears of the rotatory cuff are not so much described in the literature [4–6].

Most of the cases can be misunderstood because of being without a correct X-ray projection or the not-always clear presentation of the clinical signs, which essentially consist of pain and unability to do complete elevation and external rotation. Anyway a posterior dislocation must be suspected when there is a lesser tuberosity fracture or a fixed internal rotation of the arm.

The treatment depends on the delay of the diagnosis, on the associated bony and cartilaginous lesions, on the age and functional request of the patient, and finally on the surgeon's experience.

Most of the authors agree that a dislocation can be considered “inveterate” if it is discovered 6 weeks after the trauma. It is very important to establish if it is inveterate or not because trying to reduce an inveterate luxation can lead to an epiphyseal fracture [6–10].

The goal of the treatment of the acute posterior dislocation of the shoulder must be to restore the patient's ability to do his previous activities and to prevent posterior instability.

## 2. Case Report

We present the case of a 57-year-old man with right hemiparesis and epilepsy secondary to a stroke.

The patient presented pain and impossibility at the elevation and extrarotation of his left shoulder soon after a convulsive crisis during his first hospitalization in the Department of Neurology of our hospital.

**Figure 1:**
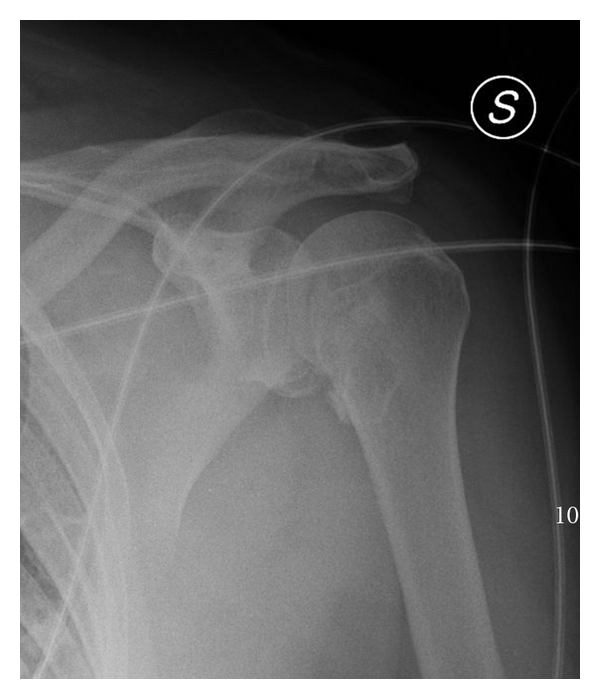
X-ray shows fracture of the humeral head and intrarotation of the arm.

An X-ray was immediately made (Figure 1). It shows humeral head fractures with some very little fragments and internal rotation of the humerus.

**Figure 2:**
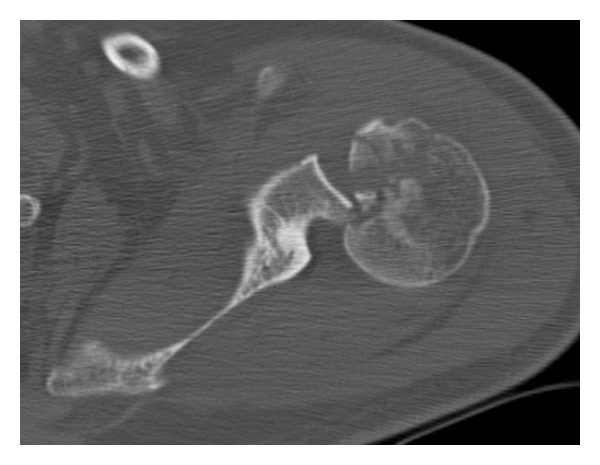
CT scan underlines the loss of cartilaginous surface and the posterior dislocation.

In order to confirm the posterior dislocation and to discover the associated fractures and the dimension of the reverse Hill-Sachs lesion, we requested a CT scan (Figure 2). It shows posterior dislocation and a damage of the cartilaginous surface of humeral head with loss of the bone that amounts between 20 and 30% of the articular surface. There are also a posterior glenoid rim fracture and a lesser tuberosity fracture.

**Figure 3:**
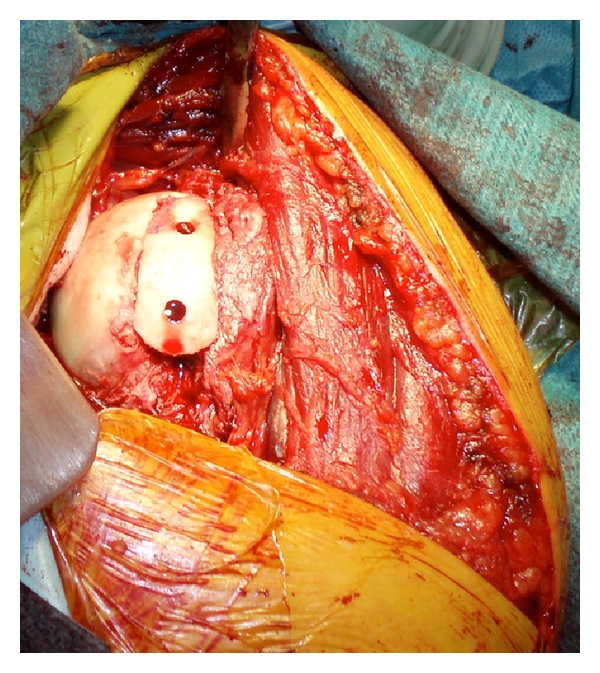
Humeral head reconstruction with allograft from crioconserved femoral head.

In order to prevent instability and to obtain an anatomical restoration that allows early mobilization and complete range of movement, we reduced the dislocation through a deltoid-pectoral incision, we stabilized the lesser tuberosity with a cancellous bone screw, and then we filled the reverse Hill Sachs with crioconserved femoral head contoured in the right shape to reconstruct the patient's humeral head, maintaining his original bending radius. The allograft was fixed by using 2 Herbert screws (Figure 3). Finally, we reduced and stabilized the posterior glenoid rim fracture with a Herbert screw, through a posterior miniapproach.

This treatment allowed the patient an early mobilization, and so he obtained a range of motion very similar to his previous condition.

**Figure 4:**
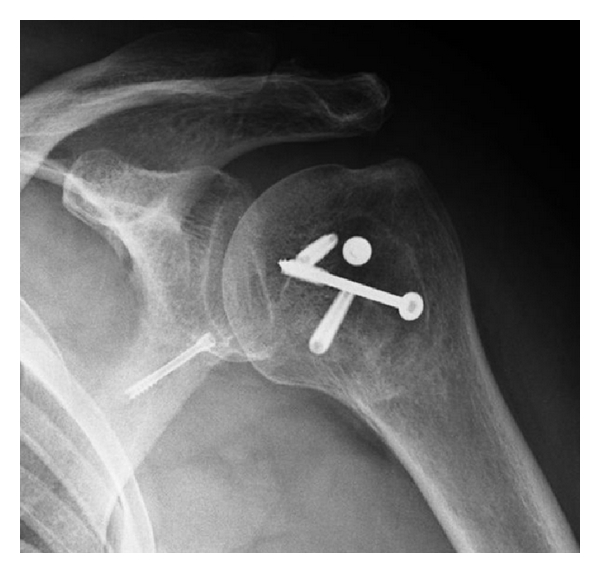
X-ray after 8 months.

**Figure 5:**
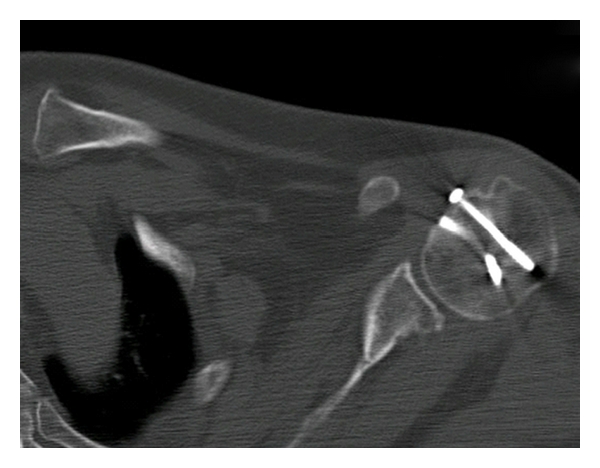
CT scan after 8 months.

**Figure 6:**
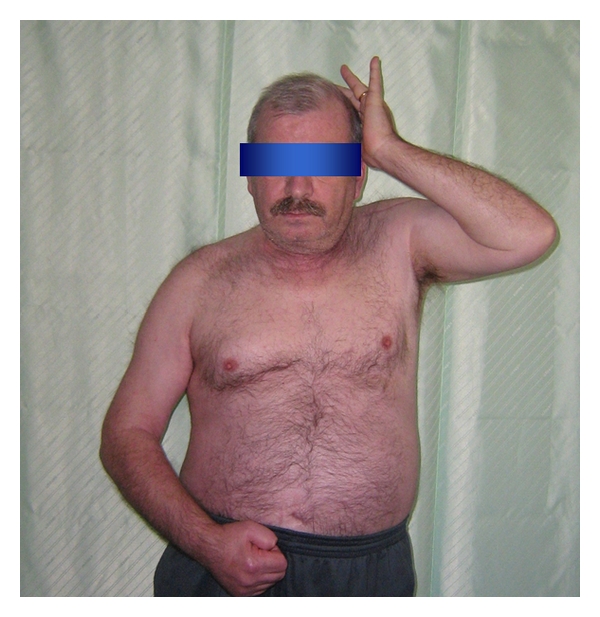
Followup after 8 months.

**Figure 7:**
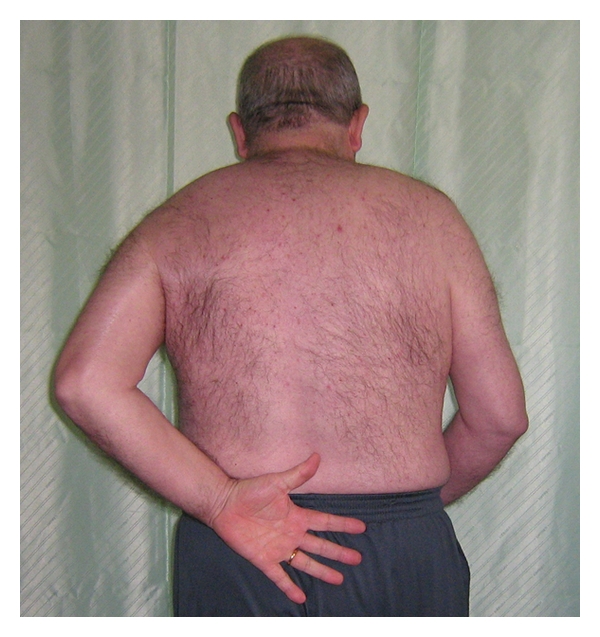
Intrarotation after 8 months.

At 8-month followup, there are no signs of avascular necrosis (Figures 4 and 5) and the patient is satisfied with our operation. We did not perform the Constant score because of the right hemiparesis. However, the patient is able to keep his left arm above his head and to rotate internally till sacral bone (Figures 6 and 7). He has no pain.

## 3. Discussion

Although the surgical techniques used for chronic posterior instability are quite the same for acute traumatic dislocations, it is important to make the right diagnosis soon after the trauma to avoid avascular necrosis [7–10]. 

Sometimes it is quite difficult to obtain correct X-ray projection because of the pain that limits the patient's mobility. A posterior dislocation must be suspected when there is a fracture of the anteromedial humeral head and when the arm is fixed in internal rotation. CT scan is always necessary to study the osteocartilaginous surface damage [5, 6].

McLaughlin first had explained the relevance of the percentage of the articular humeral head cartilage loss to decide the treatment [7, 8].

For less than 25%, Hawkins reported his outcomes of 7 cases with no treatment. As shown by Loebenberg, the decision depends on the age and on the functional request of the patient [6].

For a cartilaginous loss that is between 25 and 40%, there is no universal agreement with reduction and osteosynthesis or prosthesis, while total or hemiarthroplasty is considered necessary for damage superior to 40% [4, 7, 8].

Reconstruction of the shape of the humeral head by elevation of depressed cartilage and subchondral buttressing with cancellous bone graft has been advocated as a joint-preserving alternative [4, 5].

Dubouesset proposed the use of crioconserved allograft to restore the anatomy and to preserve the bone stock, preparing, that way, the patient for a probable future joint replacement [2]. 

Gerber and coworkers reported their study of 4 patients treated with allograft. They had excellent results in 3 cases and 1 avascular necrosis at 1-year followup [4].

Beside the case discussed here, we treated this way other 2 chronic posterior shoulder dislocations, and in both of them we achieved satisfying results, so it is our opinion that using crioconserved bone to reconstruct the right shape of humeral head is a very valid method, and it is good in prevision of a future prosthesis. When crioconserved bone is not available, it is our opinion that using autologous transplantation from iliac crest is a good solution. Maybe no delay in diagnosis can help to avoid avascular necrosis, but we believe that further studies are necessary.
